# Vertical funding, non-governmental organizations, and health system strengthening: perspectives of public sector health workers in Mozambique

**DOI:** 10.1186/1478-4491-11-26

**Published:** 2013-06-14

**Authors:** Abdul H Mussa, James Pfeiffer, Stephen S Gloyd, Kenneth Sherr

**Affiliations:** 1 Ministry of Health, Mozambique, National Malaria Control Program, Eduardo Mondlane Avenue, Maputo 1008, Mozambique; 2 Department of Global Health, School of Public Health & Health Alliance International, University of Washington, 4534 11th Avenue Northeast, Seattle, WA 98105, USA

**Keywords:** Brain drain, Africa, Health sector, Aid effectiveness, Vertical disease programming, HIV/AIDS, Ministry of health, Non-governmental organizations, PEPFAR

## Abstract

**Background:**

In the rapid scale-up of human immunodeficiency virus (HIV) care and acquired immunodeficiency syndrome (AIDS) treatment, many donors have chosen to channel their funds to non-governmental organizations and other private partners rather than public sector systems. This approach has reinforced a private sector, vertical approach to addressing the HIV epidemic. As progress on stemming the epidemic has stalled in some areas, there is a growing recognition that overall health system strengthening, including health workforce development, will be essential to meet AIDS treatment goals. Mozambique has experienced an especially dramatic increase in disease-specific support over the last eight years. We explored the perspectives and experiences of key Mozambican public sector health managers who coordinate, implement, and manage the myriad donor-driven projects and agencies.

**Methods:**

Over a four-month period, we conducted 41 individual qualitative interviews with key Ministry workers at three levels in the Mozambique national health system, using open-ended semi-structured interview guides. We also reviewed planning documents.

**Results:**

All respondents emphasized the value and importance of international aid and vertical funding to the health sector and each highlighted program successes that were made possible by recent increased aid flows. However, three serious concerns emerged: 1) difficulties coordinating external resources and challenges to local control over the use of resources channeled to international private organizations; 2) inequalities created within the health system produced by vertical funds channeled to specific services while other sectors remain under-resourced; and 3) the exodus of health workers from the public sector health system provoked by large disparities in salaries and work.

**Conclusions:**

The Ministry of Health attempted to coordinate aid by implementing a “sector-wide approach” to bring the partners together in setting priorities, harmonizing planning, and coordinating support. Only 14% of overall health sector funding was channeled through this coordinating process by 2008, however. The vertical approach starved the Ministry of support for its administrative functions. The exodus of health workers from the public sector to international and private organizations emerged as the issue of greatest concern to the managers and health workers interviewed. Few studies have addressed the growing phenomenon of “internal brain drain” in Africa which proved to be of greater concern to Mozambique’s health managers.

## Background

The rapid increase in funding for human immunodeficiency virus (HIV) care and treatment over the last 10 years has presented both great opportunities and new dilemmas for improving health service delivery in many African countries. Because this new wave of large-scale funding is primarily disease-specific, it is typically directed toward “vertical” projects through separate and parallel systems designed to improve HIV-related programs, often without strengthening other sectors of health systems [1-5]. Many donors have chosen to channel most of their funds to non-governmental organizations (NGOs) and other partners rather than to public sector systems, further reinforcing vertical approaches. However, the challenges associated with scale-up of HIV care services, especially anti-retroviral treatment (ART), has led to growing recognition that overall health system strengthening will be essential to meet acquired immunodeficiency syndrome (AIDS) treatment goals [3,6,7].

Health workforce capacity building is a vital component of health system strengthening in developing countries. It is now widely accepted that the shortage of health workers in many African countries is among the most significant constraints to achieving the three health-related Millennium Development Goals (MDGs) [8-10]. While the scale of new vertical HIV-specific funding is significantly larger than most other health sector donor funding in many African countries, its impact on existing public sector systems and workforce has been understudied [11]. There is even less in the literature that highlights the views, experiences, and working conditions of public sector health workers themselves as they attempt to manage health services within this new environment. A qualitative examination of health workers’ perspectives can complement quantitative approaches to health services research to reveal additional costs and benefits of vertical funding to health systems strengthening in Africa while suggesting further avenues of inquiry.

Since Mozambique has experienced an especially dramatic increase in disease-specific support over the last eight years it provides an ideal site to examine health worker experiences. Mozambique’s Primary Health Care (PHC) system, first established after independence in 1975, suffered through a protracted civil war and budget cuts mandated by a World Bank/International Monetary Fund (IMF) structural adjustment program during the 1980s [12]. Since the war’s end in 1992, hundreds of international NGOs and agencies have been recruited by donors and have become major actors in the health sector [13,14]. Beginning in the early 2000s, Mozambique experienced an especially large surge of aid funding primarily for HIV (and to a lesser extent malaria and tuberculosis) from donors including the President’s Emergency Plan for AIDS Relief (PEPFAR), the Global Fund to Fight AIDS, Malaria, and TB, the Clinton Presidential Foundation, the World Bank, and a range of others. Overall health sector spending increased from US$165 million in 2001 to an estimated US$591 million by 2008 [15,16]. Much of this additional funding has supported new and existing foreign NGOs leading to an expansion of their involvement in the health sector. However, even with this rapid growth in funding, the MOH continues to suffer a severe workforce shortage with a population/physician ratio of 34,579: 1, and a population/nurse ratio of 4,441:1, among the worst in the world [8]. Most support from major donors for human resources in many developing countries continues to focus on short-term in-service training rather than pre-service training that might alleviate workforce shortages [17].

Vertical funding takes two major forms in Mozambique. Some support is provided to programs within the Ministry of Health (MOH) itself that focus on specific diseases. A much larger proportion of vertical support is channeled directly to NGOs that center on specific projects whose funding, planning, and implementation are conducted outside the MOH. In both forms, vertical funds have generally not supported cross-cutting human resources, administration, logistics costs, or basic training institutions of the MOH [18,19]. “Non-vertical” aid funds that flow to the MOH itself are either channeled into the general state health budget under direct MOH control or into a mechanism known as the “Common Fund” (managed through a Sector Wide Approach to planning, or SWAp) where donor funds are jointly managed with the MOH. From 2001 to 2008 the MOH state budget doubled from US$70 million to US$138 million and the Common Fund increased from US$17 million to US$74 million. However, vertical funds channeled outside of the MOH to NGOs and other agencies quadrupled from $75 million to an estimated $300 million, accounting for over 58% of all health sector spending by 2008, while the common fund constituted about 15% and the general state budget contributed 27% [15,16]. By 2011, vertical funding still constituted nearly 50% of all health sector spending, while common fund contributions accounted for 22% [19]. The great majority of this vertical funding continues to come from PEPFAR, which had increased its support to US$269 million by 2011 [20]. None of the PEPFAR funding has been channeled through the common fund and is primarily allocated to NGO implementing partners. The Global Fund is the second largest contributor and had experimented with channeling resources through the common fund but changed strategies in July 2008 to vertical support [19]. The United States (USA) President’s Malaria Initiative (PMI) has been growing as a major vertical donor since 2007, and by 2012 was contributing about US$30 million [21]. Foreign NGOs had already been major actors in the health sector before the increase in HIV-related funding [14], but now continue to dominate the civil society involvement in the health sector in part because of the major vertical funding that has been channeled to them from PEPFAR, PMI, and other donors [18].

The primary objective of this study was to solicit and identify perspectives on vertical aid among key Mozambican public sector health managers who must coordinate, implement, and manage the myriad projects, agencies, and resource flows that the increase in vertical funding has produced amid these continued severe workforce staffing shortages. While these interviews were conducted in 2008 and are now somewhat dated they represent an important historical record to orient and guide future research. Specific aims of the project included identification of manager perspectives on the value of international aid funding and technical assistance, the impact of vertical funding on health system function, health system relationships with vertically-funded NGO projects, and the influence of vertical funding on work conditions within respondents’ respective sectors of the health system.

## Methods

Research activities were organized into three phases: 1) In order to identify aid flows to the health sector at national, provincial and district levels, researchers reviewed core MOH planning documents including the Poverty Reduction Action Plan (or PARPA), the 2008 Health Sector Strategic Plan (or PESS), the 2008 Economic and Social Plan (or PES), and routine annual reports; 2) Interviews to identify the scope of external technical assistance provided to the MOH were conducted with key directors in relevant national MOH departments including HIV/AIDS, malaria, tuberculosis, cooperation, pharmacy, human resources, and planning; and 3) Health managers at each level of the health system were then selected for in-depth qualitative interviews conducted from September to December, 2008. Data analysis and background document review was completed by 2010.

### Sample and data collection

Based on document review and preliminary interviews described above, two provinces (which remain unspecified for reasons of confidentiality) and four districts within those two provinces were chosen for inclusion in this study because of their extensive history and experience with international support, and current presence of a significant number of international partners, such as NGOs and foreign universities. A total of 42 MOH health workers/health service managers were invited for interviews, and one individual refused to participate in the study, resulting in a total of 41 who completed interviews. At each level the top directors and managers of key programs and administrative areas were invited to participate. (Programs are not identified in order to maintain confidentiality). Eight national level directors, six provincial level managers per province, and five district level managers from three districts (six in a fourth) agreed to interviews (see Table 1). The study received ethical approval from the University of Washington Institutional Review Board (UW IRB) and the Mozambique Ministry of Health National Bioethical Committee. Respondents were asked to sign consent forms approved by the UW IRB and the Mozambique Committee.

**Table 1:** Number and position of health workers interviewed

**Level**	**Number**	**Position**
National	8	Top national directors and managers
Provincial	12	Provincial directors, provincial medical chiefs, community health program managers, planning and financial managers
District	21	District directors, district doctors, pharmacy and community health program managers
**Total**	**41**	

The lead researcher used semi-structured open-ended interview guides to solicit perspectives on the value of international aid funding and technical assistance, the impact of vertical funding on health system function, health system relationships with vertically-funded NGO projects, and the influence of vertical funding on work conditions within respondents’ respective sectors of the health system. All interviews were conducted in respondents’ offices and clinics, and were recorded, transcribed, and then analyzed by the lead researcher using a theme analysis and coding approach [22], [23]. The lead researcher conducted a close reading of each transcript individually and coded them for basic important themes that emerged from the interviews. After this within-case coding process was completed, the lead researcher conducted an across-case coding procedure to identify themes shared across multiple respondents focusing on key issues mentioned consistently by majorities of the respondents. Broader codes were defined and applied to each of these themes. Together with the second author, the lead researcher created a matrix of themes and codes for further comparison and consolidation to identify overarching broad themes and subthemes articulated and shared among a majority of respondents. The lead and second authors reviewed the coded transcripts to reach consensus concerning which representative exemplar quotes to choose for presentation that best capture and express the key dimensions of each major theme and subtheme.

## Results

The interview guides were structured to elicit responses to a set of related questions focusing on the impact of vertical funding on the health system. Analysis of interview texts revealed four major themes that cut across responses to these questions and characterize the perspectives of respondents from all three levels of the health system. There were no major differences in these themes among the three health manager levels, indicating a strong consensus and similarity of experience. Themes included: 1) Advantages of vertical funding: All respondents emphasized the value and importance of international aid and vertical funding to the health sector and each highlighted program successes that were made possible by recent increased aid flows, especially HIV funding; 2) Coordination: All respondents described difficulties in coordinating external resources and challenges to local control over the use of international aid resources channeled to international NGOs; 3) Inequalities: Respondents explained how vertical funds are often channeled to specific services while other sectors remain under-resourced thus creating inequalities within the health system itself; and 4) Internal brain drain: The exodus of health workers from the public sector health system to NGOs provoked by large disparities in salaries and work conditions was mentioned by nearly all respondents (see Table 2). Within each of these themes, important subthemes were also recognized that produced a clear set of widely shared concerns. These key themes and related subthemes are elaborated further below with accompanying exemplary quotes drawn from interview texts.

**Table 2:** Organized themes and subthemes from the interviews

	**Themes**	**Subthemes**
Advantages	1. Specific support	• Infrastructure/equipment
Disadvantages	2. Leadership: power to control and coordinate external resources	• Competing agendas
		• Parallel systems
		• Accountability
		• Capacity of negotiation
	3. Inequalities within the National Health System	• Lack of integration
		• Refresher training
		• Neglected services and infrastructure
	4. Internal brain drain	• Impact of exodus from public sector to NGOs
		• Salaries and work conditions
		• Upgrading skills

### Advantages of vertical funding

Every respondent in the sample of 41 initiated their responses with a discussion of the advantages and positive results that international aid to the health sector had produced. Many respondents pointed to success in the scale-up of anti-retroviral treatment since 2004 as a major achievement made possible by international aid and the initial vertical approach it used. Others emphasized that a large proportion of the overall budget is provided by international donors and that specific projects and programs supported by vertical funds have often had significant impact (see Table 3 for summary of typical comments). However, in all interviews respondents quickly turned to the major challenges that vertical funding specifically has created for the health system.

**Table 3:** Successes of vertical funding

**Subtheme**	**Quote**
Infrastructure	“After the Peace Agreement, we had a massive support for reconstruction of the infrastructure; not only the destroyed ones but also we built new health centers and hospitals. Along with that, new equipment was put in place as well as medical supply and others…if we didn’t have International aid, nothing would have been done.” (National director)
	“[it] is true that the indicators are improving, if you look at ART, there are more than hundred thousand in treatment, we begun with about 8 thousand in 2005.” (National director)
	Source: 41 interviews of Mozambique Ministry of Health managers 2008

### Coordination, leadership, and control

According to respondents at all levels of the health system the channeling of funding to NGOs has created a series of challenges to effective management and coordination of projects, resources, and technical assistance. Coordination challenges were manifested in several key subthemes in the interviews (Table 4).

**Table 4:** Leadership, coordination, and control

**Subtheme**	**Quote**
Competing agendas	“[M]ost of the organizations that arrive in the province, they already have their own plan, they come with the kind of activities they want to implement, they come with districts already identified that they want to support, independently of what priorities the provincial directorate has.” (Provincial health manager)
	“There are partners that have their own agenda….they never discussed it with us, they simply arrive and say, I propose to work in community A, B, C with X disease preventive programs, because it’s convenient for me to work in province D, E, F. You don’t know if it’s convenient for the disease program to leave them there, or if it would be more helpful if they went to province G.” (Provincial health manager)
	“[B]ecause there were cases that in the same districts we had 2 or 3 organizations, this was always an issue of discussion, the reallocation of NGOs, because the NGOs liked to go to districts more attractive, with good transport means, water, electricity or other kinds of attractive things. And we always argued that there were prioritized districts that we should pay more attention.” (National director)
Parallel Systems	“We already have a recording and reporting system in place, that is also recommended by WHO, but there is a partner that is always asking for other information that we don’t regularly collect although we know that it’s happening in the field, it becomes difficult…so they have to introduce new forms and they are not taking into account that in some health facilities we just have a nurse that has to collect all this information in addition to his/her regular job.” (National director)
	“[D]ifferent NG0s have different methods of financial management, most of the time we end up identifying other people to manage these funds. For example, I have people to manage funds for X disease program, another person to manage funds for NGO Y, another person to manage another fund…it’s very time consuming and a lot of papers…if we had the same management fund rules it would save time and human resources.” (Provincial manager)
	“[D]uring the two years that I stayed in district M, home based care for HIV was happening only in 4 neighborhoods, I mean, 3 organizations working in the same place, we had a dispute between them, with so many other areas uncovered…it also had implications in numbers that they used to send to us for statistics…maybe their numbers are referring to the same people.” (District program manager)
Accountability	“[B]ut in terms of financial report, this is where we have a big constraint, because there are few NGOs that are transparent and openly state the budget they have for certain activities…even for district planning its difficult since you don’t know how much are you going to be supported.” (District manager)
Capacity for negotiation	“[T]his NGO is based outside Africa. Requests for funds need permission from the NGO headquarters. Well, the request leaves this province, goes to Maputo and from there to headquarters. Meanwhile there are some errors in the request and it comes back again to be corrected and after that it’s submitted again. By the time the permission arrives in this province if it was an emergency, many people would have died without the support. And then I ask, is it useful to have this NGO working with me? Is there an option for more flexibility?” (Provincial manager)
	Source: 41 interviews of Mozambique Ministry of Health managers 2008

Managers from district to national level cited the difficulties in coordinating organizations with priorities different from those of the MOH, and managing competing agendas among NGOs working in the same programmatic areas. Because many NGOs do not harmonize their priorities with the MOH, their support is difficult to coordinate, and it creates redundancies in some areas that leave other populations without needed services.

The many agencies implementing separate projects at all three levels of the system has led to creation of parallel NGO data collection, logistics, and program systems to generate rapid results and provide data needed to satisfy individual donors. The creation of duplicate information systems within the health system also adds to the workload of already overburdened staff.

Concerns about accountability of funding were raised by both national and provincial managers. One MOH officer cited the lack of NGO transparency as a major management challenge to planning and monitoring of projects. Respondents at all levels expressed frustration with partners who are intent on supporting the health sector but do not share financial or planning information openly.

Respondents highlighted the difficulty in negotiating with partners over use of resources in the health sector; negotiation and leadership skills are needed, many stated, to explain the gaps in services and priorities in ways that partners understand.

### Inequalities in the national health system created by vertical funding

Some vertical funding is provided directly to MOH activities and is targeted to improve specific programs within the MOH, such as HIV testing, malaria bed nets, or TB programs. The imbalances created within the health system itself can be disruptive, respondents said (Table 5). Vertical funding can undermine integration of primary health care services through lack of attention to the systems and processes that cut across programs and help integrate them. Several respondents noted the increase in ART patients in a short period has dramatically increased the number of clinic files with no support for improving basic filing systems and skills. Vertical funds have not supported training for basic cross-cutting administrative systems necessary to properly follow HIV patients.

**Table 5:** Vertical funding and inequalities within national health system

**Subtheme**	**Quote**
Lack of integration	“In vertical programs, the results are immediate…if you invest in Malaria you will see the results in a short time, it’s good for those who want to see the impact of funding but when you talk about the National Health Service, this is bad, since the resources are allocated for specific areas and we are not sorting out the problems of the communities.” (National director)
	“The importance people give to these three diseases [HIV, malaria and TB] is a detriment to, for example, mother and child health, diarrhea, environmental health or mental health…I think that we should integrate all programs, and we would have better results.” (Provincial manager)
	“We should look at the system, because if the partners are saying that they are giving funds to improve the health of population, then they should improve health in general and not choose specific areas, because what affects population health is a set of factors, a range of diseases that already exist, so if we look at one disease, we will help in that specific disease but the global result, to improve health, will not happen because there will be a lot of gaps.” (Provincial manager)
Refresher training	“[M]ost funding for ongoing training comes from vertical programs, and it’s natural that the programs have an interest in investing in people who perform such activities, they are not interested in funding ongoing training for all the workers.” (District manager)
	“[F]or example, in a health center, if you talk with the health workers, some will tell you that in the last very few years they had 10 training courses and you will find out that 8 or 9 are HIV-related courses.” (Provincial manager)
	“[B]ecause these [vertical] programs have a lot of funds, they absorb all existing capacity for ongoing training, and unfortunately in planning we never plan ongoing training per se.” (Provincial manager)
Neglected services and infrastructure	“No one has ever thought of strengthening the capacity of archiving the clinical files…That’s where we often lose patient information.” (National director)
	“[T]hen we buy equipment [for a vertical program], where will this equipment be placed? Under a tree? It’s necessary to fund infrastructure, as well electricity, water in order for that equipment to work until we achieve the final goal, to test that patient with that specific disease. Well, we can’t think about strengthening the health system if all these basic conditions are not satisfied.” (National director)
	“Well, if we talk about infrastructure, you will see that this laboratory, I think that this doesn’t have either good equipment nor good infrastructure. Because we have support from NGO “A”, we have a good laboratory outside this building where we perform all HIV patients’ analysis.” (District director)
	Source: 41 interviews of Mozambique Ministry of Health managers 2008

Vertical funds support refresher training strategies that often focus exclusively on skills related to single diseases or health programs without supporting management skills to improve cross-cutting administrative systems. According to several human resource managers, nurses not linked with vertical programs may go without refresher trainings of any kind for more than 10 years after graduating. Other nurses may repeat retraining courses many times simply because they work in a specific disease area.

District managers stated that imbalances in support for specific programs or areas within the same province has led some health units to have regular drug stockouts while NGO support to other units mitigates stock ruptures. The NGO support should be broad and province-wide, provincial and district managers stated, and not specific to one health unit, district, or catchment area. Differences in infrastructure support have provided among the most visible imbalances in the health system. New HIV service facilities are often new and well equipped, while other program service facilities remain deteriorated they claimed.

### Internal brain drain

The exodus of health workers from the public system represented the single greatest problem with vertical funding approaches, according to virtually all the managers interviewed. It was perceived as a widespread and persistent problem causing lasting damage to the integrity of the national health system, and represents a net loss to the system’s long term investment in human resources. Higher salaries, better work conditions, and more rewarding opportunities for career development all contributed to pulling health workers from the system according to interviewees (Table 6). The effect of this internal brain drain on the public sector is seen as widespread and long lasting. The health workers who leave for NGOs are often recruited because they are good workers with strong experience, and their departures sometimes leave gaps in the national health system that are difficult to fill.

**Table 6:** Internal brain drain

**Subtheme**	**Quote**
Impact of exodus from public sector to NGOs	“The big problem we had and still have with NGOs, is the competition for resources, human resources, they steal almost all human resources we have. Most NGOs, although they have expatriates they also have a lot of national staff, and that makes imbalances in the system.” (Provincial director)
	“[I]t’s a profound loss for the health sector, as most of the time, they [human resources] are key elements in the system, with long experience and sometimes they are essential staff…the NGOs are more unlikely to contract workers who are recent graduates.” (Provincial manager)
Salary and work conditions	“People leave the national system and go to NGOs, international agencies and private sector just because they are looking for better salaries.” (National director)
	“[I] have received proposals from organizations to leave the national system. First, the salary proposed was 3 or 4 times higher. Besides the salaries, they were giving me other incentives like a house, transport and others. So you start making your short term plan and realize that if you stay in public sector you will not have a chance to meet those short term goals.” (District manager)
	“[T]he difference is huge, if I work for an NGO and have the job like I have here, I would earn probably US$ 2000, and here I don’t earn more than US$ 300, you can see the difference? US$ 1700. It’s a lot.” (Provincial manager)
Upgrading skills	“[S]ometimes we have someone learning monitoring and evaluation skills with a partner, but when he/her is trained to perform the job, he/she leaves the national system and surprisingly the same partner will offer them a job.” (Provincial manager)
	“For example, in this province, the only chance to study at university is to do management and pedagogy courses. Because my goal is to upgrade my skills, I can work for an NGO and make more money and pay the school fees so I can move to another NGO….I have many colleagues doing Biology, others are doing management.” (Provincial manager)
	Source: 41 interviews of Mozambique Ministry of Health managers 2008

According to respondents, high NGO salaries are the primary pull factor. Differences in work conditions beyond salary were also cited by health system managers as key motives to leave the public system. In some cases technical assistance training within the system leads to health workers seeking employment with their new skills outside the health system. The opportunity to upgrade skills and advance one’s career was also cited by respondents as a major incentive for health workers to consider working for an NGO. The additional savings from NGO employment are apparently often used by some to finance training for more lucrative careers outside the health sector entirely.

## Discussion

The respondents in these interviews emphasized a great appreciation for the support provided to the health sector by donors and NGOs, and were quick to cite many of the successes achieved in recent years with the help of vertical funding. However, there was broad consensus among respondents at all levels of the system that increased funds for specific health programs have brought major new challenges in coordination of externally-funded projects, imbalances within health system, and loss of key personnel to vertically funded NGOs. The difficulties in coordinating the many projects implemented by the rapidly increasing number of NGOs in the health sector are compounded by each agency’s preferences for specific geographical areas and special projects [24].

Mozambique endorsed the 2005 Paris Declaration on Aid Effectiveness and is a signatory of the International Health Partnership (IHP+) in 2007 [25]. Both initiatives were intended to support harmonization of aid through country-led national health strategies that lead to better donor coordination and accountability. The ongoing difficulties in coordinating vertical funding reported by respondents in this study suggest continuing challenges in aid harmonization in Mozambique. Indeed, a 2012 IHP+ update states: 

“The 2012 IHP+Results report finds that, despite the endorsement of various aid management frameworks, there is much less progress in the ways aid is actually delivered in country. Countries appear to have moved further than development partners on putting the IHP+ principles into practice. Altogether, development agencies have met only 3 of the 12 targets for effective health aid reported by IHP+Results” [26].

In Mozambique, the MOH has repeatedly attempted to coordinate all agencies in the health sector by innovating new coordination tools and emphasizing MOH priorities for both disease programs as well as broader system support [27]. The SWAp process was introduced as an additional policy to join the partners together with the MOH in setting priorities, harmonizing planning, and coordinating support. Nevertheless, the proportion of overall health sector funding channeled through the SWAp process has varied annually between 14% and 22% from 2008 to 2011 and has been dwarfed by vertical funding that has contributed between 45 and 58% over that period [19]. The findings from this study suggest that these imbalances have made it difficult for key MOH staff to effectively coordinate efforts and therefore strengthen programs. Management skills become weaker among staff when moving from central to peripheral level making coordination of external resources even more difficult at lower levels in the health system, according to respondents.

While it is beyond the scope of the current study to examine why these challenges persist, the continued large scale funding dedicated to specific diseases may contribute to these coordination difficulties. The preference of major donors such as PEPFAR and PMI to channel funds to NGOs and away from the common fund may further contribute to coordination complexity reported here. The flow of funds into the MOH for disease-specific projects creates challenges for managers since other critical, often administrative sectors, remain unsupported. Respondents repeatedly cited imbalances in training as additional funding for disease-specific trainings diverted scarce administrative resources from trainings in other crucial support sectors.

The exodus of health workers from the public sector to international NGOs and other agencies emerged as the issue of greatest concern to the managers and health workers interviewed for this study. Brain drain has been described in many African countries, but primarily in relationship to health workers migrating abroad [8]. Few studies have addressed the growing phenomenon of “internal brain drain”, which is of greater concern to Mozambique’s health managers according to the findings from this study [28]. The expansion of support for NGOs and other international partners through vertical funding has created a much larger job market and provoked increased health worker recruitment by NGOs from the public sector according to respondents. A 2009 study in Mozambique collected data on salary disparities and documented 5- to 10-fold salary differentials between public sector and NGO staff at equal rank in Mozambique [29]. Of the 723 Mozambican physicians trained between 1980 and 2006, 25% had left the public sector. This rate may appear somewhat low compared to other neighboring nations, however Mozambique’s private health sector has remained very small and language barriers limit external migration in comparison to nearby English-speaking countries. Among those leaving the public sector for employment within Mozambique (N= 113), 66.4 percent worked for NGOs and 21.2 percent for bilateral and multilateral donors [30]. By 2010, over 58 percent of internal migration cases (N = 66/113) worked for organizations financed primarily by PEPFAR.

There are important limitations to this study and to the interpretation of its findings. The study was intended to capture the perspectives of health system workers specifically and therefore only represents their impressions and experiences. It was beyond the scope of the study to interview donors, NGOs and international agencies to provide alternative views and representations. While there was striking unanimity among the respondents in terms of the key themes described here, the sample of 41 does not represent all key managers in the national health system and there may be greater variation in the broader health worker population. Also, the themes and views presented in this study represent impressions and subjective experiences within a complex environment. It is also possible that those who were interviewed include many who were unable to leave the public sector for better pay elsewhere and therefore offer a more negative view of external assistance. However, the views reported here were so widely shared and consistently expressed that it is unlikely that this aspect of their experience would account for the general perspectives offered. Further validation through other data sources would be required to draw definitive conclusions.

The following recommendations follow from these findings and should be considered to address the key challenges posed by international aid and vertical funding provided to the health sector in Mozambique: 1) A similar study should be conducted with the international partners to compare their perspectives on the utility and effectiveness of vertical programs and aid coordination; 2) More studies are needed to measure internal brain drain and evaluate its impact on public sector services, and to identify policies that the MOH and donors can adopt to reduce the loss of health workers; 3) Donors should consider greater investment in leadership and management to provide public sector health workers with skills and negotiating power to better control external resources; 4) Together with the MOH of Mozambique, partners should consider innovative ways to use vertical funding for health system strengthening; and 5) NGOs and donors should consider practices that support the strengthening of public sector human resources and capacity building and avoid practices that contribute to internal brain drain.

## Conclusions

Major donors such as PEPFAR will likely continue to channel a great majority of their funding to NGOs. Significant portions of funding to the MOH itself will continue to be earmarked for specific programs causing potential systemic imbalances. While disease-specific funding is welcome and appreciated, health managers interviewed here suggest that the better integration of vertical programs into primary health care systems combined with a shift to more financing for administration and recurrent costs that support basic operations can strengthen the health system. Leadership training for MOH cadres should be emphasized since more coordination and negotiation capacity is needed in the MOH to harmonize all stakeholders to integrate services. The MOH should consider creating a package of incentives together with higher salaries to retain qualified workers.

## Abbreviations

AIDS: Acquired immune deficiency syndrome; ART: Anti-retroviral treatment; HIV: Human immunodeficiency virus; MDGs: Millennium development goals; MOH: Ministry of health; NGOs: Non-governmental organizations; PARPA: Action program for the reduction of absolute poverty; PEPFAR: President’s emergency plan for AIDS relief; PES: The 2008 Economic and Social Plan; PESS: The 2008 Health Sector Strategic Plan; PHC: Mozambique’s primary health care system; PMI: President’s Malaria Initiative; SWAp: Sector wide approach to planning.

## Competing interests

The authors are all public sector employees, but otherwise have no competing interests.

## Authors’ contributions

AM helped conceptualize the study and study design, collected and analyzed the data, and wrote the manuscript. JP helped conceptualize the study, analyze data, write the first draft, and edit subsequent drafts. KS helped conceptualize the study and edit subsequent drafts. SG helped conceptualize the study and edit subsequent drafts. All authors read and approved the final manuscript.
